# Is Mitral Valve Repair Superior to Mitral Valve Replacement in Elderly Patients? Comparison of Short‐ and Long‐Term Outcomes in a Propensity‐Matched Cohort

**DOI:** 10.1161/JAHA.116.003605

**Published:** 2016-07-28

**Authors:** Miriam Silaschi, Sanjay Chaubey, Omar Aldalati, Habib Khan, Mohammed Mohsin Uzzaman, Mrinal Singh, Max Baghai, Ranjit Deshpande, Olaf Wendler

**Affiliations:** ^1^ Department of Cardiothoracic Surgery and CardiologyKing's College HospitalLondonUK

**Keywords:** mitral valve, surgery, survival, Catheter-Based Coronary and Valvular Interventions, Cardiovascular Surgery

## Abstract

**Background:**

Because of demographic changes, a growing number of elderly patients present with mitral valve (MV) disease. Although mitral valve repair (MV‐repair) is the “gold standard” treatment for MV disease, in elderly patients, there is controversy about whether MV‐repair is superior to mitral valve replacement. We reviewed results after MV surgery in elderly patients treated over the past 20 years.

**Methods and Results:**

Our in‐hospital database was explored for patients who underwent MV surgery between 1994 and 2015. Survival data, obtained from the National Health Service central register, were complete for all patients. Of 1776 patients with MV disease, 341 were aged ≥75 years. Patients with repeat cardiac surgery, endocarditis, and concomitant aortic valve replacement were excluded. This yielded 221 MV‐repair and 120 mitral valve replacement patients. Concomitant procedures included coronary artery bypass grafting in 135 patients (39.6%) and tricuspid valve surgery in 50 patients (14.7%). Thirty‐day mortality was 5.4% (MV‐repair) versus 9.2% (mitral valve replacement, *P*=0.26). Overall 1‐ and 5‐year survival was 90.7%, 74.2% versus 81.3%, 61.0% (*P*<0.01). Median survival after MV‐repair was 7.8 years, close to 8.5 years (95% CI: 8.2–9.4) in the age‐matched UK population (ratio 0.9). Rate of re‐operation for MV‐dysfunction was 2.3% versus 2.5% (mitral valve replacement, *P*=1.0). After propensity matching, patients after MV‐repair still had improved survival at 1, 2, and 5 years (93.4%, 91.6%, 76.9% versus 77.2%, 75.2%, 58.7%, *P*=0.03).

**Conclusions:**

Excellent outcomes can be achieved after MV surgery in elderly patients. Long‐term survival is superior after MV‐repair and the re‐operation rate is low. MV‐repair should be the preferred surgical approach in elderly patients.

## Introduction

In patients with degenerative mitral valve (MV) disease, MV repair (MV‐repair) is the generally accepted “gold standard” treatment, as it has demonstrated superiority over MV replacement (MVR) in various clinical settings.^1^ The role of MV‐repair for functional (ischemic) MV regurgitation is still under debate^2^, ^3^ and in rheumatic disease mainly MVR is performed, as MV‐repair is more complex in these patients and less durable.^4^ Due to the demographic changes in Western communities and a higher incidence of MV disease in the elderly population,^5^ the age of patients referred for MV surgery is increasing. Although there is consensus that even in elderly patients surgical treatment should be offered,^6^ there remains discussion of whether MV‐repair provides the same advantages as in younger cohorts.^7^

Data obtained from administrative American databases revealed a low rate of MV‐repair in elderly patients of <50% and identified higher age as an independent predictor of MVR.^8^, ^9^ Surgeons who perform MV operations in elderly patients need to keep operative times short, limit the complexity of procedures, and avoid the need for re‐operation, which may explain why they more often prefer to replace the MV in these patients. Moreover, the benefit of improved long‐term survival after MV‐repair is often believed not to apply in patients whose remaining life expectancy is naturally limited to less than 10 years. Published data on long‐term outcomes in elderly patients are contradictory, and the cohorts of MV‐repair and MVR in these reports often were not comparable.^7^, ^10^, ^11^, ^12^ While European and American guidelines in general favor MV‐repair over MVR, if a durable repair can be achieved, European guidelines do not address the elderly population with MV disease,^13^ and American guidelines recommend MV‐repair in patients >65 years only in the presence of symptomatic primary MV disease.^14^

Therefore, although outcomes after MV surgery in elderly patients have improved markedly during recent years,^6^, ^9^, ^12^ the question of which surgical technique should be used remains unclear. In addition, innovative catheter‐based technologies increasingly address MV disease^15^ and should be measured against outcomes of conventional treatment options. In this investigation we analyze outcomes after MV‐repair and MVR in elderly patients at our institution.

## Methods

### Patient Data

**Figure 1:**
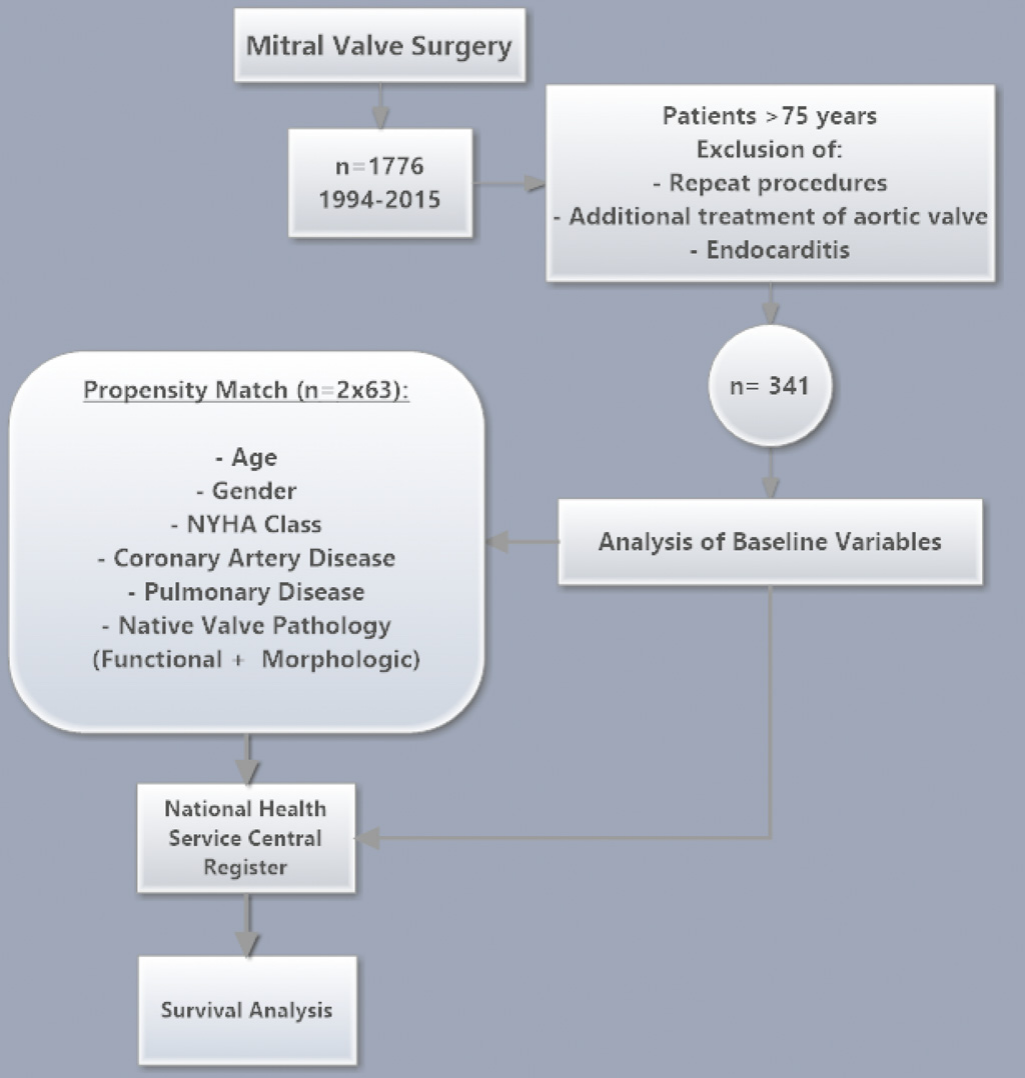
Study design. NYHA indicates New York Heart Association.

We retrospectively explored our dedicated in‐hospital database for patients who underwent MV surgery between 1994 and 2015. Of 1776 patients treated, those aged <75 years, or who underwent concomitant aortic valve replacement, surgery of the aorta, repeat cardiac procedures, or suffered from active endocarditis were excluded. According to the treatment received, the remaining 341 patients were divided into a cohort of MV‐repair (n=221) and MVR (n=120) and were compared regarding all documented baseline variables. Variables with significant difference between the groups were included for propensity matching (threshold *P*=0.10). This yielded 126 patients after MV‐repair (n=63) and MVR (n=63). Both cohorts, unmatched and matched, were included in this study for further analyses (Figure 1).

Valve etiology and lesions were determined by the surgeon during invasive inspection of the valve. Degenerative pathology was defined as leaflet prolapse due to chordal elongation or rupture; functional mitral regurgitation (MR) was present when asymmetrical left ventricular dilatation led to ring dilatation and displacement of the papillary muscle due to cardiomyopathy. Rheumatic valve pathology was defined as reduced leaflet motion in systole and diastole associated with leaflet thickening and commissural fusion. The decision in favor of or against MV‐repair was at the discretion of the operating surgeon, but generally MV‐repair has been the preferred treatment at our institution. MV‐repair was always associated with annuloplasty, while various repair techniques for the valve itself were used. In case of MVR, the subvalvular apparatus was preserved as per surgeon's preference; however, at least the posterior leaflet was preserved if anatomically possible and tissue valves were implanted in all cases.

Data collection of baseline and perioperative data was prospective, as part of our dedicated in‐hospital database and completeness of data set was secured by a data manager throughout the observational period. Data collection and audit was approved by the institutional R&D committee and every patient consented to data collection prior to operation. Survival data were obtained via the National Health Service central register and were complete for all patients. Data from outpatient visits and re‐admissions to our institution were retrospectively obtained by exploration of the electronic patient records.

### Primary and Secondary End Points

The primary end point was long‐term survival. Deaths were considered to be cardiovascular in the presence of a proximate cardiac cause, any extracardiac vascular cause, procedure‐related death (ie, caused by any complication of the procedure), sudden death, or death of unknown cause. Median survival of the age‐ and sex‐matched general UK population was obtained from the office for national statistics and the Eurostat data from the European Commission (www.ons.gov.uk, http://ec.europa.eu/eurostat/web/population-demography-migration-projections/deaths-life-expectancy-data/database) and compared to our study cohort. In addition, a time‐related, combined end point was created, defined as time to one of the following events: death, re‐operation for MV dysfunction or any observation of moderate or severe MR on echocardiography. In this context, the word “re‐operation” refers to any kind of re‐intervention on the MV, including transcatheter valve‐in‐valve implantation. The secondary end points were acute perioperative complications, New York Heart Association Class (NYHA) at 6 weeks, re‐admission for Heart Failure (HF), LV‐function during follow‐up, and grade of MR at 1 year. Re‐admission for HF was counted if there was a re‐admission in the time period between the initial discharge and the first outpatient visit, which is routinely performed at 6 weeks postsurgery at our institution. To strengthen analysis of follow‐up data, data available for left ventricular (LV) function at 3 and 12 months were compared to the baseline data only from those individuals for whom follow‐up was available.

### Statistical Analysis

All data obtained were retrospectively analyzed. Data are presented as absolute numbers and percentages for categorical variables and as mean values and standard deviation for continuous variables unless stated otherwise. Dichotomous variables were compared by Fisher's exact test and χ^2^ test wherever suitable and continuous variables by paired and unpaired *t* tests wherever suitable. *P*‐values were reported without correction for multiple testing. A level of significance was set to 2‐tailed *P*<0.05. A logistic regression analysis on behalf of significantly different covariates (Figure 1, threshold for logistic regression: *P*<0.1) was performed to calculate the probability of allocation to either MV‐repair or MVR. This was used as a propensity score to create homogeneous study groups. For matching algorithm, nearest‐neighbor method with an accepted maximum difference of 10% between matched individuals was applied. Analysis of baseline variables was then repeated to check for balance.

No methods were used to account for any possible correlation that may arise between the 2 groups. For survival analyses, Kaplan–Meier calculations were performed and groups were compared on behalf of the Gehan–Breslow–Wilcoxon method to account for early deaths due to the naturally limited life expectancy among this age group and the length of the observational period. Median follow‐up was calculated with the Shemper and Smith method. All statistical analyses were performed by M.S. and reviewed independently by S.C. All computation was carried out using the statistical softwares GraphPad Prism 5.0 (GraphPad Software Inc, La Jolla, CA) and SPSS 19.0 (IBM, Armonk, NY).

## Results

### Baseline Characteristics

In the overall cohort (n=341), patients undergoing MV‐repair were significantly younger (*P*=0.03), were more frequently in NYHA class I/II (*P*=0.01), and were more likely to have pulmonary disease and coronary artery disease (*P*=0.01 and <0.01, respectively). Left ventricular end‐diastolic diameter was significantly higher in MV‐repair (55.3±6.8 mm versus 50.8±7.0 mm, *P*<0.01). Patients undergoing MV‐repair more often also had a history of prior myocardial infarction (*P*<0.01) and differed significantly with regard to the underlying valve pathology. They mainly presented with pure MV regurgitation (98.6% versus 66.7%, *P*<0.01) and the majority had degenerative MV disease (66.1% versus 48.4%, *P*<0.01) (Table 1).

**Table 1:** Baseline Characteristics of the Overall Patient Cohorts

Variable	MV‐Repair (n=221)	MVR (n=120)	*P* Value
Age, y, mean±SD	79.2±3.0	78.4±2.9	0.03
Sex (female), n (%)	95 (43.0)	62 (51.7)	0.07
NYHA class			0.01
I, n (%)	16 (7.2)	5 (4.2)	
II, n (%)	94 (42.5)	33 (27.5)	
III, n (%)	81 (36.7)	61 (50.8)	
IV, n (%)	20 (9.0)	17 (14.2)	
Unknown, n (%)	10 (4.5)	4 (3.3)	
LV function, n (%)			0.17
>50%	115 (52.0)	77 (64.2)	
30% to 50%	84 (38.0)	36 (30.0)	
<30%	18 (8.1)	7 (5.8)	
Pulmonary disease, n (%)	38 (17.2)	9 (7.5)	0.01
CAD (VD, mean±SD)	1.0±1.3	0.5±0.9	<0.01
No. of VD (%)			<0.01
None	124 (56.1)	82 (68.3)	
1‐VD	22 (9.9)	19 (15.8)	
2‐VD	26 (11.8)	8 (6.7)	
3‐VD	49 (22.2)	9 (7.5)	
Unknown	0	2 (1.7)	
Prior MI, n (%)	47 (21.3)	10 (8.3)	<0.01
Prior PCI, n (%)	6 (2.7)	2 (1.7)	0.72
Diabetes, n (%)	24 (10.9)	14 (11.7)	0.86
Hypertension, n (%)	139 (62.9)	63 (52.5)	0.07
Prior stroke/TIA, n (%)	17 (7.7)	16 (13.3)	0.13
Carotid artery stenosis, n (%)	6 (2.7)	6 (5.0)	0.36
Extracardiac arteriopathy, n (%)	26 (11.8)	9 (7.5)	0.36
Operative priority			0.44
Elective, n (%)	149 (67.4)	84 (70.0)	
Urgent, n (%)	59 (26.7)	27 (22.5)	
Emergent, n (%)	5 (2.3)	5 (4.2)	
Unknown, n (%)	8 (3.6)	4 (3.3)	
Native valve pathology			<0.01
Degenerative, n (%)	146 (66.1)	59 (48.4)	
Rheumatic, n (%)	7 (3.2)	51 (41.8)	
Functional, n (%)	53 (23.9)	6 (4.9)	
Mixed, n (%)	5 (2.3)	1 (0.8)	
Unknown, n (%)	8 (3.6)	5 (4.0)	
Hemodynamic pathology			<0.01
Stenosis, n (%)	0	7 (5.8)	
Regurgitation, n (%)	218 (98.6)	80 (66.7)	
Mixed, n (%)	3 (1.4)	33 (27.5)	

CAD indicates coronary artery disease; LV, left ventricular; MI, myocardial infarction; MV, mitral valve; MVR, mitral valve replacement; NYHA, New York Heart Association Class; PCI, percutaneous coronary intervention; TIA, transitory ischemic attack; VD, vessel disease.

After propensity matching (n=126), baseline characteristics between the cohorts were balanced, as there were no significant differences measured (Table 2). Mean age was 79.1±3.1 in MV‐repair versus 78.8±3.3 in MVR (*P*=0.58) and in both cohorts, 96.8% of patients had pure MV regurgitation (*P*=1.0). Left ventricular end‐diastolic diameter was not significantly different, being 53.7±7.1 mm in MV‐repair and 51.7±6.4 mm in MVR (*P*=0.21). Moreover, most patients had degenerative MV disease (84.2% versus 76.2%, *P*=0.52), although other pathologies were still present after matching.

**Table 2:** Baseline Characteristics of the Propensity‐Matched Cohorts

Variable	MV‐Repair (n=63)	MVR (n=63)	*P* Value
Age, y	79.1±3.1	78.8±3.3	0.58
Sex (female, %)	20 (31.7)	20 (31.7)	1.00
NYHA class			0.97
I (%)	2 (3.2)	2 (3.2)	
II (%)	21 (33.3)	19 (30.1)	
III (%)	30 (47.6)	30 (47.6)	
IV (%)	9 (14.3)	10 (15.9)	
Unknown (%)	1 (1.6)	2 (3.2)	
LV function, n (%)			0.36
>50%	41 (65.1)	43 (68.3)	
30% to 50%	20 (31.7)	15 (23.8)	
<30%	2 (3.2)	5 (7.9)	
Hypertension, n (%)	41 (65.1)	37 (58.7)	0.47
Diabetes, n (%)	7 (11.1)	6 (9.5)	1.00
Pulmonary disease, n (%)	5 (7.9)	5 (7.9)	1.00
Prior PCI, n (%)	0	2 (3.2)	0.50
Previous MI, n (%)	4 (6.3)	8 (12.7)	0.36
CAD, no. of VD, n (%)			0.63
1VD	9 (14.3)	13 (20.6)	
2VD	4 (6.3)	5 (7.9)	
3VD	3 (4.8)	5 (7.9)	
Carotid artery stenosis, n (%)	2 (3.2)	5 (7.9)	0.44
Extracardiac arteriopathy, n (%)	7 (11.1)	4 (6.3)	0.53
Operative priority, n (%)			0.26
Elective	40 (63.5)	41 (65.1)	
Urgent	21 (33.3)	15 (23.8)	
Emergent	1 (1.6)	5 (7.9)	
Unknown	1 (1.6)	2 (3.2)	
Hemodynamic pathology			1.00
Stenosis, n (%)	0	0	
Regurgitation, n (%)	61 (96.8)	61 (96.8)	
Mixed n (%)	2 (3.2)	2 (3.2)	
Native valve pathology			0.52
Degenerative, n (%)	53 (84.2)	48 (76.2)	
Rheumatic, n (%)	5 (7.9)	6 (9.5)	
Functional, n (%)	4 (6.3)	5 (7.9)	
Mixed functional/degenerative, n (%)	0	1 (1.6)	
Unknown, n (%)	1 (1.6)	3 (4.8)	

CAD indicates coronary artery disease; MI, myocardial infarction; MV, mitral valve; MVR, mitral valve replacement; NYHA, New York Heart Association Class; VD, vessel disease.

### Acute Operative Results

Cardiopulmonary bypass times (MV‐repair: 101.1±35.9 minutes, MVR: 107.4±64.5 minutes, *P*=0.26) and aortic cross‐clamp times (ACC) (MV‐repair: 70.9±22.9 minutes, MVR: 74.6±23.8, *P*=0.18) were not significantly different between the 2 groups and in the propensity‐matched cohorts with cardiopulmonary bypass times of 97.2±31.2 minutes (MV‐repair) and 115.9±83.2 minutes (MVR, *P*=0.10) and aortic cross‐clamp times of 71.4±24.0 minutes (MV‐repair) and 75.0±22.8 minutes (MVR, *P*=0.40).

Patients undergoing MV‐repair were more likely to receive concomitant coronary artery bypass graft (CABG) (n=97/43.9% versus n=38/31.7%, *P*=0.03); however, there was no significant difference regarding additional procedures after propensity matching (Table 3).

**Table 3:** Acute Procedural Results and Follow‐Up After MV‐Repair and MVR

Variable	All Patients			Propensity‐Matched Cohorts		
	MV‐Repair (n=221)	MVR (n=120)	*P* Value	MV‐Repair (n=63)	MVR (n=63)	*P* Value
Bypass time, minutes, mean±SD	101.1±35.9	107.4±64.5	0.26	97.2±31.2	116±83.2	0.10
Cross‐clamp time, minutes, ±SD	70.9±22.9	74.6±23.8	0.18	71.4±24.0	75.0±22.8	0.40
Additional CABG, n (%)	97 (43.9)	38 (31.7)	0.03	14 (22.2)	23 (36.5)	0.11
Additional TVR, n (%)	33 (14.9)	17 (14.2)	1.00	11 (17.5)	8 (12.7)	0.62
Postop stay, days, ±SD	10.1±7.3	12.3±12.2	0.04	10.2±7.5	13.6±14.9	0.10
Need for IABP, n (%)	9 (4.1)	3 (2.5)	0.55	1 (1.6)	3 (4.8)	0.62
Postop hemofiltration, n (%)	29 (13.1)	23 (19.2)	0.16	8 (12.7)	12 (19.0)	0.34
Stroke/TIA, n (%)	3 (1.4)	6 (5.0)	0.07	0	4 (6.3)	0.12
30‐day mortality, n (%)	12 (5.4)	11 (9.2)	0.26	2 (3.2)	8 (12.7)	0.05
Follow‐up results						
Re‐admission for HF, n (%)	17 (7.7)	6 (5.0)	0.50	7 (11.1)	2 (3.4)	0.16
Re‐operation^a^, n (%)	5 (2.3)	3 (2.5)	1.0	2 (3.2)	1 (1.6)	1.00
Endocarditis, n (%)	0	1 (0.8)	0.36	0	1 (1.6)	1.00

Median follow‐up was 1320 days. No statistical methods were used to account for correlation that arises from propensity matching. CABG indicates coronary artery bypass grafting; HF, heart failure, any re‐admission for worsening of symptoms or procedure‐related complications occurring between discharge and first outpatient visit 6 weeks postprocedurally; IABP, intraaortic balloon pump; MV, mitral valve; MVR, mitral valve replacement; TIA, transient ischemic attack; TVR, tricuspid valve replacement.

a Any re‐operation for mitral valve dysfunction during the observational period.

Conversion of planned MV‐repair to MVR after invasive inspection of the MV occurred in 18.3% of MVR (n=22). MV‐repair was attempted but converted to MVR during 1 cardiopulmonary bypass in 2.5% (n=3), and conversion during a second cardiopulmonary bypass graft but within the index procedure in 0.8% (n=1). Comparison of 30‐day mortality after “unplanned MVR” versus “planned MVR” yielded no significant difference (n=2/7.7% versus n=9/9.6%, *P*=1.00). One of the patients with conversion after attempted repair died on day 2 due to a LV rupture with subsequent pericardial tamponade. Postoperative length of stay was shorter after MV‐repair in the unmatched cohort (10.1±7.3 days versus 12.3±12.2 days, *P*=0.04) but although still shorter, not significantly different after propensity matching (MV‐repair: 10.2±7.5 days, MVR: 13.6±14.9 days, *P*=0.10). Acute in‐hospital complications were not significantly different, as shown in Table 3. Mortality at 30 days was not significantly different before propensity matching (MV‐repair: n=12/5.4%, MVR: n=11/9.2%, *P*=0.26), but was significantly lower after MV‐repair in the matched cohorts (n=2/3.2% versus n=8/12.7%, *P*=0.05). In the propensity‐matched cohort, deaths after MV‐repair were due to severe sepsis in 1 patient and hypoxic brain injury after cardiac arrest in a second patient. After MVR, 4 patients died of perioperative congestive heart failure with low cardiac output. One additional patient had a postoperative myocardial infarction with subsequent heart failure. From the remaining 2, 1 patient had multiorgan failure after a pericardial tamponade and another had multiorgan failure after LV rupture with extensive surgical repair. In 1 patient after MVR, cause of death at 30 days was unknown.

### Symptoms 6 Weeks Postsurgery and Re‐Admission for Heart Failure

Between discharge from the index procedure to the first outpatient visit at 6 weeks, re‐admission for HF occurred in 7.7% (n=17) after MV‐repair versus 5.0% (n=6) after MVR, which was not significantly different (*P*=0.50) and did not change after the cohorts were propensity matched (MV‐repair: n=7/11.1%, MVR: n=2/3.4%, *P*=0.16).

Of all survivors, 75.7% (n=237) attended the surgical outpatient clinic 6 weeks after surgery and were questioned with regard to exertional dyspnea. Patients of both cohorts had improved significantly in NYHA class compared to the same individuals at baseline (*P*_trend_<0.01 for MV‐repair and MVR, respectively). In the MV‐repair cohort, 80.2% were in NYHA class I/II at 6 weeks, compared to 80.0% in the MVR cohort (*P*=1.0).

### Survival

**Figure 2:**
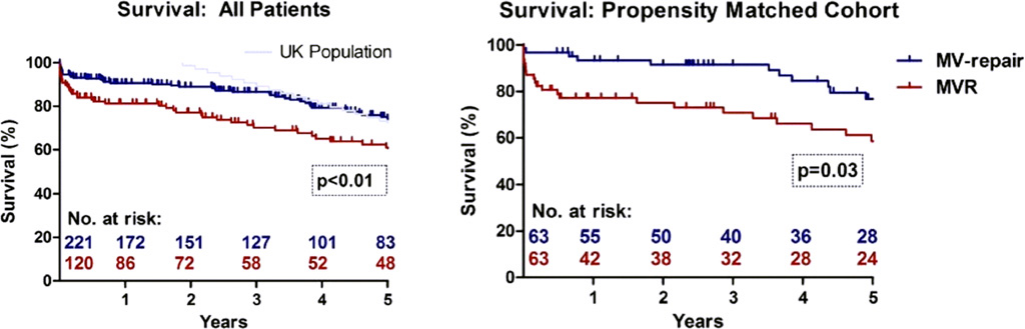
Long‐term survival after MV‐repair and MVR in the overall and propensity‐matched cohorts. MV indicates mitral valve; MVR, mitral valve replacement.

Exploration of the National Health Service central register facilitated 100% completeness of survival data. Median follow‐up was 5.3 years (1920 days) in the overall cohort, being 5.1 years in MV‐repair (1849 days) and 6.7 years in MVR (2443 days). Long‐term survival of the total cohort was superior after MV‐repair with Kaplan–Meier survival at 1, 2, and 5 years of 90.7%, 89.0%, and 74.2% versus 81.3%, 77.1%, and 61.0% (*P*<0.01). In patients with degenerative MV pathology, survival at 1 and 5 years was 95.0% and 81.8% (MV‐repair) versus 81.8% and 64.9% (MVR, *P*=0.02). This survival benefit was still seen after propensity matching with 1‐, 2‐, and 5‐year survival of 93.4%, 91.6%, and 76.9% versus 77.2%, 75.2%, and 58.7% (*P*=0.03). Cause of death was identified in 95.6% of deaths at 30 days (22/23) and 73.8% of deaths at 12 months (31/42). At 12 months, deaths were noncardiovascular in 5.0% in MV‐repair (1/20) and 9.1% in MVR (2/22, *P*=1.00). Comparison to the age‐ and sex‐matched population showed that patients who survived the first year after MV‐repair had similar life expectancy as the general UK population (Figure 2). The yearly mortality rate of 4.1% after the first year in the MV‐repair cohort corresponds to the natural yearly mortality of UK citizens aged 80 years of 4.9% in 2013 as reported by the European Commission and the office for National Statistics. Yearly mortality after MVR was 5.1%.^16^ Median life expectancy after MV‐repair was 7.8 years versus 8.5 years of age‐ and sex‐matched UK citizens (ratio: 0.9).

### Event‐Free Survival

During follow‐up, re‐operations for MV dysfunction were performed in 5 cases of the MV‐repair cohort (2.3%) after a mean time of 181.8±122.7 days. In 4 of these patients, the indication was a failure of the repaired MV (1.8%) with recurrent MR: 1 patient presented with MV stenosis attributable to calcific degeneration of the repaired valve. This patient underwent transcatheter valve‐in‐ring implantation and made an uneventful recovery. In the MVR cohort, re‐operation for MV‐dysfunction occurred in 3 cases (2.5%), due to structural deterioration of the implanted tissue valves. Re‐operation rates were not significantly different between the cohorts before and after matching (*P*=1.0). However, time to re‐operation was significantly shorter after MV‐repair with 181.8±122.7 and 2544±1445 days, respectively (*P*<0.01). All patients who had a re‐operation survived the procedures and at least the first 2 years afterwards (mean survival: 5.5 years).

Significant MR (moderate/severe) was observed in 15 cases after MV‐repair (6.8%) after a mean time period of 838.1±1063 days; in 4 of these cases, re‐operation was performed as mentioned above. After MVR, significant MR was observed in 3 cases (2.5%) after a mean period of 2087±1964 days. All of these were due to structural valve degeneration and were re‐operated. Rate of significant MR during the observational period was not significantly different in the unmatched (*P*=0.13) and matched cohorts (*P*=0.11). There was 1 patient with suspicious MV endocarditis observed in the MVR cohort (0.8%) after 92 days; this patient was treated successfully with antibiotic medication.

**Figure 3:**
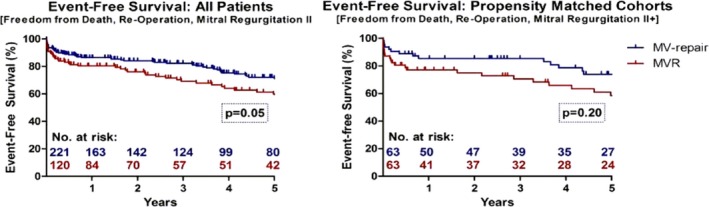
Event‐free survival after MV‐repair and MVR in the overall and propensity‐matched cohorts. MV indicates mitral valve; MVR, mitral valve replacement.

As a result, Kaplan–Meier comparison of event‐free survival was significantly different in the unmatched cohort (*P*=0.05) but not significantly different between the propensity‐matched cohorts (*P*=0.20, Figure 3).

### Mitral Regurgitation and LV Function at 1 Year

Data on severity of MR at 1 year postsurgery were available in 127 cases (47.7% of survivors) and are visualized in Figure S1. Grade of MR at 1 year was significantly different after MV‐repair (*P*<0.01), with 6.5% of patients (6/92) demonstrating moderate or severe MR. In addition, “mild to moderate” MR was observed in 7.6% of patients (7/92) and “mild” MR in 39.1% (36/92). In contrast, MVR patients presented with moderate or severe MR in 2.9% (1/35), “mild to moderate” in 2.9% (1/35), and “mild” in 17.1% (6/35).

Data on LV function were available in 148 cases either at 3 or 12 months postsurgery (55.6% of survivors) and are summarized in Figure S2. Direct individual correlation, which included only data of the same individuals at baseline and follow‐up, showed no significant change at 3 months after MV‐repair and MVR, respectively (*P*_trend_=1.00 and 0.63), but showed significant improvement in LV function after MV‐repair at 12‐month follow‐up, as the proportion of patients with a normal LV ejection fraction of >50% increased from 50.0% to 76.0% (*P*_trend_=0.04). After MVR, individuals presented with the same LV function at 12 months compared to baseline (*P*_trend_=0.78). Left ventricular end‐diastolic diameter at 3 months showed improvement from 56.6±6.5 to 50.4±7.0 mm in MV‐repair (*P*<0.01) and from 51.0±0.7 to 48.2±8.2 mm in MVR (*P*=0.03). In patients with 12‐month follow‐up, LV end‐systolic volume index improved from 34.2±14.1 to 29.2±12.7 mL/m^2^ after MV‐repair (mean change from baseline −4.9 mL/m^2^, *P*_trend_=0.03) and remained stable after MVR, being 30.2±14.4 mL/m^2^ at baseline and 27.3±15.2 mL/m^2^ at 12 months (mean change from baseline −2.9 mL/m^2^, *P*_trend_=0.57).

## Discussion

Due to demographic changes in the Western world and the high prevalence of MV disease among the aging population, elderly patients are increasingly referred for MV treatment. Although for young patients it is accepted that MV‐repair is the preferred surgical technique with respect to improved outcomes, this is still discussed for elderly patients. In addition, the range of available treatment options for MV disease is broadening rapidly towards interventional strategies to repair or replace the MV in an attempt to reduce the trauma of surgery and to improve outcomes, particularly in the elderly group of patients.^15^ However, future techniques to be introduced into clinical practice will have to be measured against established surgical outcomes.

In this analysis, we provide information for these 2 areas of interest by analyzing outcome data of MV surgery and comparing outcomes of MV‐repair and MVR in an elderly patient cohort, which in addition to the previously published literature, can be used for future comparison with interventional treatments.

### Baseline Characteristic

Preoperative risk of our study cohort was elevated as most patients were in their late 70s and a considerable number of patients had a history of pulmonary‐ and peripheral vascular disease, stroke, and LV dysfunction. As a retrospective calculation of established risk scores (eg, logistic EuroSCORE) was not possible, due to missing variables required for assessment, comparisons to published cohorts are made on the basis of single variables. Compared to the cohort of a randomized trial comparing percutaneous MV‐repair with surgery, patients of our cohort less often had concomitant coronary artery disease but were more than 10 years older.^17^ Compared to a meta‐analysis of outcomes after MV surgery in octogenarians, the risk profile of our cohort is similar.^18^^.^

### Acute Operative Results

Age has previously been identified as an independent predictor of MVR.^8^ The low threshold to replace the MV in elderly patients is often triggered by the view of some surgeons that MV‐repair is more complex, takes longer ischemic time and risks to result in recurrent MR. In our cohort, MV‐repair did not result in longer aortic cross‐clamp and bypass times compared to MVR. Operation times can be kept short in the hands of experienced surgeons, which obviously reduces the cardiac and operative trauma. Nevertheless, even in our center—specialized in MV‐repair—conversion to MVR after initial valve inspection happened in 18.3%, reflecting the preference of some surgeons for MVR if MV‐repair is expected to be complex. These patients presented mainly with pathologies such as extensive bileaflet or anterior leaflet prolapse, and myxomatous degeneration with extensive tissue and leaflet calcification. The factor “age” also accounted for some of the conversions. Given our experience and the results of this investigation, the decision against MV‐repair should be based only on anatomical factors and feasibility of MV‐repair and not on the age of a patient. While the overall rate of successful MV‐repair for degenerative MV disease since 2004 was 80.6% (441/547) at our center, it was 91.0% (143/158) in our most experienced surgeon (*P*<0.01). Correspondingly, MV‐repair was performed in 87.9% (116/132) in ischemic MV disease compared to 100% (64/64) in the most experienced surgeon (*P*<0.01). This also emphasizes that patients in whom MV‐repair is seen to be complex should be managed by experienced and specialized surgeons to increase the likelihood of a successful MV‐repair with minimal operative trauma.

### Symptoms 6 Weeks Postsurgery and Re‐Admission for Heart Failure

Symptom relief at 6 weeks of follow‐up was equally achieved in both our cohorts as reflected by NYHA class. The rate of re‐admission for heart failure during this period was equally low. We conclude that both procedures are clinically effective in the short term. Unfortunately, long‐term data on valve‐related symptoms were not available for our cohort as patients were not followed up systematically. Nevertheless, a rate of >90% long‐term efficiency of MV‐repair in degenerative disease has been widely reported.^19^^.^

### Survival

In our experience, short‐ and long‐term survival was superior after MV‐repair. The higher 30‐day mortality of MVR patients, also confirmed in the propensity‐matched cohort, is a strong argument to repair the MV even in elderly patients. This is even more impressive as significantly more patients with MV‐repair underwent concomitant coronary artery bypass graft in the unmatched cohort. A higher short‐term mortality after MVR as compared to MV‐repair has been described widely for patients of all age groups,^1^ although selection bias cannot be fully excluded in the studies presented. As the majority of deaths seen after MVR at 30 days were due to heart failure with low cardiac output syndrome, it seems possible that altering LV geometry by MVR may acutely affect survival and thus lead to a higher operative mortality.

Nevertheless, the burden of MV surgery is better reflected by 6‐month mortality as most deaths occur during the first 6 months.^7^ Chikwe and colleagues reported a propensity‐adjusted analysis of procedural outcomes after MV surgery in octogenarians, in which operative mortality with 11.0% for MV‐repair and 18.9% for MVR was higher compared to our cohort. However, they included patients with endocarditis (1.8% in MV‐repair and 13.7% in MVR) and had a higher rate of patients with ischemic MV disease (32.2% in MV‐repair) as compared to our cohort. The mean age of the study population was 83 years, which is higher than in our cohort and may also explain the difference in outcomes.^12^ Vassileva and colleagues showed a lower operative mortality in elderly patients of 3.9% for MV‐repair and 8.9% for MVR, but they reported only on outcomes of isolated MV procedures.^8^ Given our results, we can add that MV surgery combined with coronary artery bypass graft or tricuspid valve replacement can also be performed with low mortality in the elderly population.

Survival at 5 years in a large retrospective analysis of outcomes after MV surgery was 57%, compared to 74% after MV‐repair in our cohort.^6^ In their analysis, rate of MV‐repair was 71% and baseline characteristics were similar. However, the study cohort was historical with patients treated between 1980 and 1995. Given the overall increased life expectancy as well as the above‐described improved peri‐operative management in cardiac surgery, it should have led to more favorable outcomes currently. In fact, the authors were able to show the improvement of outcomes over time in their study population, as operative mortality decreased from 27% to 5% in elderly patients. However, they did not make a direct comparison of MV‐repair to MVR.^6^ Another reason for improved outcomes in our cohort might be the routine preservation of the full or at least posterior subvalvular apparatus. This could also explain the superior long‐term survival of our cohort compared to 20% to 30% survival at 5 years observed for octogenarians in a recent meta‐analysis.^18^ Of note, the majority of studies included in this meta‐analysis consisted of historical cohorts from the 1990s. In fact, the authors themselves state that the date of publication was linked to survival differences. Andalib and colleagues conclude on the basis of a pooled 30‐day mortality rate for MV‐repair of 6% that MV surgery may be associated with poor outcomes and uncertain benefit for quality of life in these patients.^18^ We strongly disagree with that conclusion, especially when made for MV‐repair. They recommend an increasing use of interventional treatment in elderly patients on basis of their data, without further comment on actual 30‐day mortality of currently established interventional treatments (4% to 14%).^20^ The very low 30‐day mortality of MV‐repair in our cohort (5.4% overall and 3.2% without coronary artery bypass graft) should be taken as an objection. Therefore, we agree with others that age alone should not preclude consideration of MV‐repair.^8^ On the other hand, the high operative mortality reported for MVR should draw attention to the important ongoing development of an increasing number of interventional treatment options for the native MV. They should serve as a useful alternative in cases when successful MV‐repair is unlikely or perioperative mortality is expected to be high. The discussion of the patient's condition and various treatment opportunities should be held by the multidisciplinary “Heart Team.” Given that approximately one half of patients with symptomatic MV disease are not referred for surgery because they are deemed too high risk,^21^ these meetings should not only be used to determine clinical treatment, but are also ideal to assess and consider new interventional treatment options.^22^

### Event‐Free Survival

In contrast to the belief that the rate of recurrent MR is high in elderly patients with calcified and frail tissue, the rate of significant MR in our cohort was low after MV‐repair at 1‐year follow‐up, but may be underestimated due to incomplete echo data. Although the rate of recurrent MR was higher after MV‐repair, it also did not translate into a higher rate of re‐operation or mortality, as the rate of re‐operation for valve dysfunction during the observational period was identical between the cohorts. The improved survival may be triggered by the significantly improved LV ejection fraction and LV end‐systolic volume index after MV‐repair at 1‐year follow‐up. Furthermore, life with a valve prosthesis may be an additional risk factor for adverse survival in MVR patients. However, the majority of patients in our analysis had degenerative mitral valve pathology. Fundamentally, the etiology of MV disease dictates repair feasibility and success. In a recently published, randomized controlled trial comparing MVR and MV‐repair for ischemic MV disease, there was no difference in the mortality rate between these procedures, but there was a higher incidence of recurrent MR after MV‐repair.^2^ The rate of recurrent MR and repeat surgery was very high in their multicenter study with only small groups of patients included from numerous institutions. In our study, with mainly degenerative pathologies included, survival was strongly in favor of MV‐repair despite a higher rate of recurrent MR. In the propensity‐matched cohort, event‐free survival was not significantly different, due to the higher rate of recurrent MR in the MV‐repair group. However, actual re‐operation rates were low and correspond to observations made in a randomized controlled trial, published by Feldman and colleagues.^17^

These findings must be considered when assessing outcomes of interventional treatment options. While transcatheter mitral valve‐in‐valve implantation for failed prostheses or rings in mitral position in high‐risk patients is a promising concept, interventional treatment of the native MV, especially in patients with degenerative MV disease, proves to be more challenging. The most established interventional treatment for native MR is the MitraClip^™^ therapy (Abbott, Menlo Park, CA), which imitates the surgical technique of the Alfieri stitch by building a bridge between the anterior and posterior mitral leaflet. However, recent reports of MitraClip^™^ therapy observed a rate of recurrent severe MR of around 55% at 12 months,^17^ while re‐operations for recurrent MR were reported as >20% at 4 years of follow‐up in patients treated for degenerative MR.^23^ Interestingly, a substantial number of patients, initially considered unsuitable for surgical treatment and accepted for MitraClip^™^ therapy, underwent MV surgery later on. In patients who undergo surgical “Alfieri procedures,” the combination of valvuloplasty and annuloplasty is believed to create a durable and successful MV‐repair. The missing component of annuloplasty in MitraClip^™^ procedures may therefore be one cause of recurrent MR. Moreover, MitraClip^™^ therapy can complicate future MV‐repair, as valves after failed MitraClip^™^ procedures are often not repairable, especially if more than one clip has been placed.^24^ We therefore conclude that elderly patients with degenerative MR and acceptable surgical risk should undergo surgery as the initial “gold standard” treatment. Transcatheter mitral valve‐in‐ring implantation might still be an option should a repaired valve fail, provided that the implanted ring is not dehiscent and adaptable to circular shape.^25^

### LV Function

The MV plays a fundamental role in the structural and functional integrity of the LV. The anterior MV leaflet is believed to act like a rudder during systole, supporting blood flow through the LV outflow tract. The chordae tendineae prevent ventricular dilatation, and discontinuation of this geometry results in progressive LV stress.^15^ Correspondingly, we observed improved LV function after MV‐repair, despite a higher degree of residual MR. Although LV function post MVR did not deteriorate, as described previously,^26^, ^27^ which may be a result of preservation of the subvalvular apparatus, LV function did not improve, despite optimal function of the replaced valves. Possibly, future strategies to implant a MV interventionally may overcome this problem as the entire subvalvular apparatus will be preserved with less lateral chordal displacement. At present, transcatheter MVR strategies for native MV disease are still complicated by the risk of LV outflow tract obstruction due to radial forces of the transcatheter heart valves, risk of valve migration, and paravalvular leakage due to higher systolic stress and weaker anchoring opportunity within a less calcified mitral annulus.

## Conclusions

In an elderly, propensity‐matched patient cohort, survival after MV‐repair is superior to MVR. Moreover, MV‐repair is associated with a low re‐operation rate and recurrent severe MR. This survival benefit may be due to the improvement in LV function seen after MV‐repair over a period of 12 months, while LV function was unchanged after MVR. We therefore conclude that in elderly patients, the same guidelines for choosing operative strategies for treating MV disease should be applied based on the underlying pathology, irrespective of a patients' age. In degenerative MV disease, MV‐repair should be the preferred surgical treatment given the increasing use of interventional techniques to treat mitral regurgitation in this elderly group of patients. The data of this investigation and others should be used as a benchmark for future outcomes of these new techniques.

### Limitations

Although propensity matching was performed to account for differences between the study cohorts, a retrospective analysis of results may still be biased, as propensity scores only balance measurable confounders; unmeasured confounders still occur and may bias treatment analyses. Due to the limited number of patients available, patients with different MV pathologies were included for analyses, which may weaken the conclusions made from its results. While 100% completeness of survival data is a strength of our analysis, patients were not followed up systematically and clinical data were available only for a part of the study population as specified in the respective sections of this article. In addition, observation of a patient population over a period of more than 20 years may be biased by improvements in surgical techniques over time, change in population characteristics, and different skills of surgeons. Although we have accounted for time of surgery in a logistic regression analysis, the effect of such a long observational period on outcomes cannot be eliminated completely. Symptoms of patients in the long term could not be assessed.

## Sources of Funding

Dr Silaschi's research post is funded through the King's College Hospital Charity.

## Disclosures

None.

## Supporting information


**Figure S1.** Grade of MR 1 year after MV‐repair and MVR (*P*<0.01). Data were available for 127 patients (47.4% of survivors). MV indicates mitral valve; MVR, mitral valve replacement.
**Figure S2.** Direct correlation of LV function in individuals with available follow‐up. LV indicates left ventricular.
