# Supplementary material for: Is Mitral Valve Repair Superior to Mitral Valve Replacement in Elderly Patients? Comparison of Short‐ and Long‐Term Outcomes in a Propensity‐Matched Cohort
Source: J Am Heart Assoc. 2016 Jul 28;5(8):e003605. doi: 10.1161/JAHA.116.003605 (PMC5015286; doi:10.1161/JAHA.116.003605)

SUPPLEMENTAL MATERIAL

Figure S1: Grade of MR 1 year after MV-repair and MVR (p<0.01). Data was available for 127 patients (47.4% of survivors).

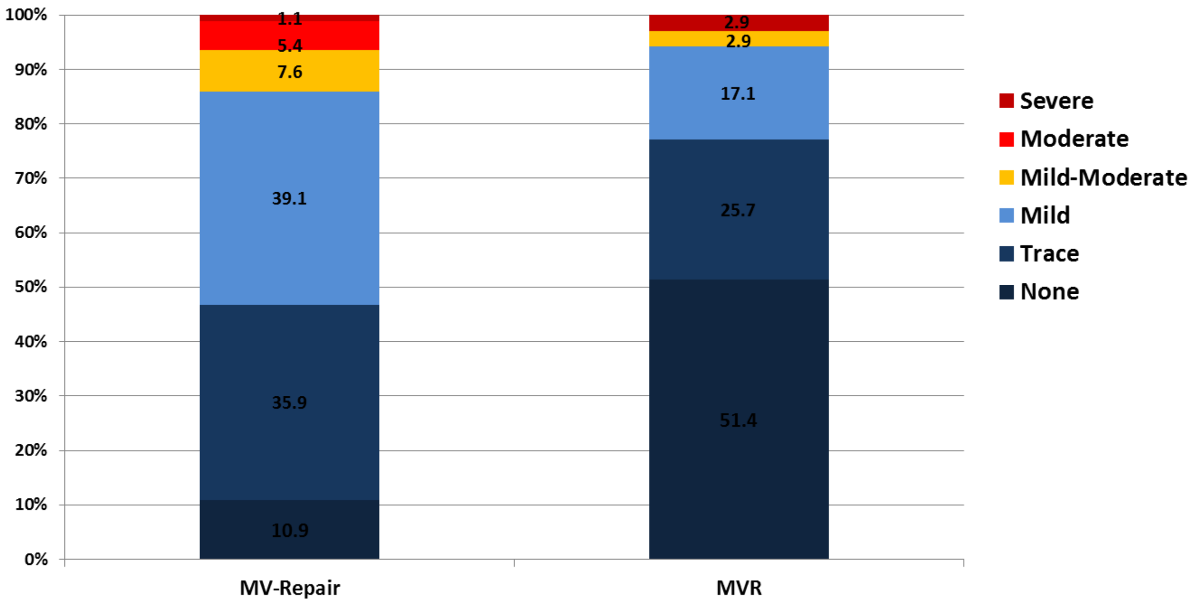

Figure S2: Direct correlation of LV-function in individuals with available follow-up.

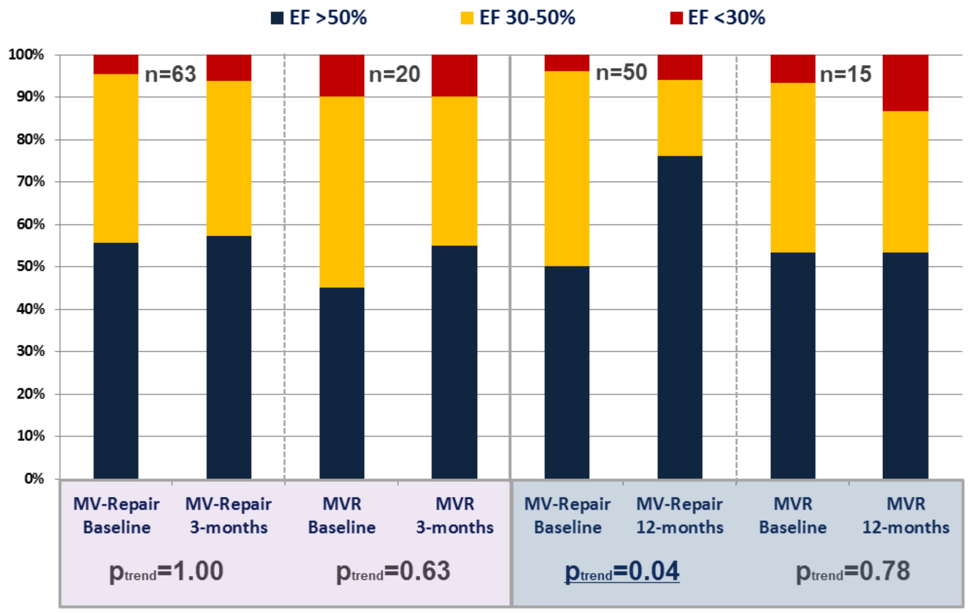

Supplement: Supplementary file 1 — Figure S1. Grade of MR 1 year after MV‐repair and MVR (P<0.01). Data were available for 127 patients (47.4% of survivors). MV indicates mitral valve; MVR, mitral valve replacement. Figure S2. Direct correlation of LV function in individuals with available follow‐up. LV indicates left ventricular. [file JAH3-5-e003605-s001.pdf]
